# Imputing Variants in HLA-DR Beta Genes Reveals That HLA-DRB1 Is Solely Associated with Rheumatoid Arthritis and Systemic Lupus Erythematosus

**DOI:** 10.1371/journal.pone.0150283

**Published:** 2016-02-26

**Authors:** Kwangwoo Kim, So-Young Bang, Dae Hyun Yoo, Soo-Kyung Cho, Chan-Bum Choi, Yoon-Kyoung Sung, Tae-Hwan Kim, Jae-Bum Jun, Young Mo Kang, Chang-Hee Suh, Seung-Cheol Shim, Shin-Seok Lee, Jisoo Lee, Won Tae Chung, Seong-Kyu Kim, Jung-Yoon Choe, Swapan K. Nath, Hye-Soon Lee, Sang-Cheol Bae

**Affiliations:** 1 Department of Rheumatology, Hanyang University Hospital for Rheumatic Diseases, Seoul, Republic of Korea; 2 Division of Rheumatology, Department of Internal Medicine, Kyungpook National University School of Medicine, Daegu, Republic of Korea; 3 Department of Rheumatology, Ajou University School of Medicine, Suwon, Republic of Korea; 4 Division of Rheumatology, Daejeon Rheumatoid & Degenerative Arthritis Center, Chungnam National University Hospital, Daejeon, Republic of Korea; 5 Division of Rheumatology, Department of Internal Medicine, Chonnam National University Medical School and Hospital, Gwangju, Republic of Korea; 6 Division of Rheumatology, Department of Internal Medicine, Ewha Womans University School of Medicine, Seoul, Republic of Korea; 7 Division of Rheumatology, Department of Internal Medicine, Dong-A University, Busan, Republic of Korea; 8 Division of Rheumatology, Department of Internal Medicine, Arthritis & Autoimmunity Research Center, Catholic University of Daegu School of Medicine, Daegu, Republic of Korea; 9 Arthritis and Clinical Immunology Research Program, Oklahoma Medical Research Foundation, Oklahoma City, Oklahoma, United States of America; University of Alabama at Birmingham, UNITED STATES

## Abstract

The genetic association of *HLA-DRB1* with rheumatoid arthritis (RA) and systemic lupus erythematosus (SLE) is well documented, but association with other HLA-DR beta genes (*HLA-DRB3*, *HLA-DRB4* and *HLA-DRB5*) has not been thoroughly studied, despite their similar functions and chromosomal positions. We examined variants in all functional HLA-DR beta genes in RA and SLE patients and controls, down to the amino-acid level, to better understand disease association with the HLA-DR locus. To this end, we improved an existing HLA reference panel to impute variants in all protein-coding HLA-DR beta genes. Using the reference panel, HLA variants were inferred from high-density SNP data of 9,271 RA-control subjects and 5,342 SLE-control subjects. Disease association tests were performed by logistic regression and log-likelihood ratio tests. After imputation using the newly constructed HLA reference panel and statistical analysis, we observed that *HLA-DRB1* variants better accounted for the association between MHC and susceptibility to RA and SLE than did the other three HLA-DRB variants. Moreover, there were no secondary effects in *HLA-DRB3*, *HLA-DRB4*, or *HLA-DRB5* in RA or SLE. Of all the HLA-DR beta chain paralogs, those encoded by *HLA-DRB1* solely or dominantly influence susceptibility to RA and SLE.

## Introduction

HLA-DR is a key molecule implicated in conferring risk for rheumatoid arthritis (RA), systemic lupus erythematosus (SLE) and other diseases. HLA-DR is present on the surface of antigen-presenting cells as heterodimers consisting of an alpha chain (HLA-DRα; encoded by *HLA-DRA*) and a beta chain (HLA-DRβ; encoded by *HLA-DRB1*, *HLA-DRB3*, *HLA-DRB4*, or *HLA-DRB5*) [1]. HLA-DRβ has variable coding variations especially in its peptide-binding groove, in contrast to the beta chain proteins [1].

Recently, genetic studies have fine-mapped the primary association within the major histocompatibility complex (MHC) locus with RA and SLE to *HLA-DRB1*, and further narrowed it down to specific amino-acid positions [2–4]. However, these studies did not investigate the other functional HLA-DR beta genes (*HLA-DRB3*, *HLA-DRB4*, or *HLA-DRB5*) due to the lack of a reference panel suitable for imputing their genetic variants, although all the HLA-DRB genes are in strong linkage disequilibrium and encode beta chains functionally the same as HLA-DRβ1. It is thus very important to examine the associations of all the HLA-DRB genes simultaneously with *HLA-DRB1*-associated diseases.

Here, we constructed an HLA reference panel [5] to impute all functional HLA-DRB genes and dissected their associations with RA and SLE to better understand HLA-DR association with these diseases.

## Materials and Methods

### Genotyping *HLA-DRB3*, *HLA-DRB4*, and *HLA-DRB5* to construct an HLA reference panel

413 unrelated Korean individuals were genotyped for four-digit classical HLA alleles and for copy numbers of *HLA-DRB3*, *HLA-DRB4*, and *HLA-DRB5* using a Roche GS 454 sequencing system at the Institute for Immunology and Infectious Diseases (IIID; Murdoch WA, Australia) and IIID's institution-specific calling algorithms that were accredited by the American Society for Histocompatibility and Immunogenetics (ASHI). These 413 individuals are the same individuals who were used in a previous Korean HLA reference panel [5]. The analysis was approved by the Institutional Review Board of Hanyang University, and written consent was obtained from the participants.

### Constructing a Korean HLA reference panel for imputation

The previous Korean HLA reference panel had haplotype-level data of 2- and 4-digit classical alleles and amino acid residues of 6 HLA genes: *HLA-A*, *HLA-B*, *HLA-C*, *HLA-DRB1*, *HLA-DPB1* and *HLA-DQB1*, from 413 unrelated Korean individuals [5]. In this study, we additionally merged the data for copy number, classical allele, and amino-acid residue of *HLA-DRB3*, *HLA*-*DRB4*, and *HLA*-*DRB5* of the same 413 Korean subjects with the existing data in the previous HLA reference panel [5]. Amino-acid residues were defined based on the amino-acid sequence of 4-digit alleles in the IMGT/HLA database [6]. In the panel, a bi-allelic marker was encoded as allele *A*/*B*, and a multi-allelic marker was encoded as presence/absence for each allele of the marker. The HLA-DR locus houses zero or one gene copy of *HLA-DRB3*, *HLA-DRB4*, and *HLA-DRB5*, which allowed us to encode the information on copy number as presence/absence of each gene in a haplotype.

The new HLA imputation reference panel was constructed by phasing 5,858 MHC SNPs and amino acid residues, 2-digit and 4-digit HLA alleles, and copy number of 9 HLA genes using the Beagle 3.0.4 imputation program [7] powered by the SNP2HLA method [8] with some modifications.

### Imputing HLA variants in Korean case-control cohorts

We extracted MHC SNPs with minor allele frequency ≥1% from previous genome-wide and Immunochip SNP datasets [4,9] of Korean case-control cohorts for RA (n = 9,271; 2,234 cases and 7,036 controls; 2 independent cohorts) and SLE (n = 5,342; 849 cases and 4,493 controls; 1 cohort). Information on these cohorts and data has been presented in previous reports [4,9]. The study involving case-control participants was approved by the Institutional Review Board of Hanyang University, and written consent was obtained from the participants. Datasets for each cohort were used to impute HLA variants by SNP2HLA [8] and the new Korean HLA reference panel. Imputed markers with minor allele frequency ≥1% and imputation quality (PLINK INFO) ≥0.8 were used in disease association tests.

### Testing disease association

Disease association at each imputed marker was tested by logistic regression, adjusting the top 10 principal components (PCs) calculated from genome-wide SNP data [4,9]. The disease association of each amino-acid position with multiple residues was examined by log-likelihood ratio tests [4,9]. The null logistic regression model included only the top 10 PCs as predictors, and the full model additionally included the dosage of the tested markers, excluding the most frequent allele. In addition, because the datasets for RA were separately imputed from two different array datasets (a genome-wide SNP array and an immunochip array dataset), we used a dummy variable indicating datasets in fitting a logistic regression model.

## Results and Discussion

We improved an existing HLA reference panel [5] to additionally impute copy numbers (0 to 2 copies for each gene), classical alleles, and amino-acid residues of *HLA-DRB3*, *HLA-DRB4* and *HLA-DRB5*, as well as variants in pre-existing HLA target genes. In brief, the three HLA-DRB genes were genotyped by sequencing 413 Korean subjects who were used in a previous Korean HLA reference panel [5]. Long-range haplotypes were then constructed by phasing the HLA-DRB variants with all pre-existing variants of SNP and HLA (*HLA*-*A*, *HLA*-*B*, *HLA*-*C*, *HLA*-*DRB1*, *HLA*-*DPB1*, and HLA-*DQB1*).

Imputation accuracy in imputation using the new HLA reference panel was examined by cross-validation comparing the imputed and actual genotypes of classical alleles of *HLA*-*A*, *HLA*-*B*, *HLA*-*C*, *HLA*-*DRB1*, *HLA*-*DPB1*, *HLA*-*DQB1*, *HLA*-*DRB3*, *HLA*-*DRB4* and *HLA*-*DRB5*, as described following. The Korean HLA reference panel subjects (n = 413) were randomly divided into 10 almost equal sized subgroups (n≈41). Of the 10 subgroups, a single subgroup (n≈41) is retained as the test group to be imputed for HLA variants, and the remaining 9 subgroups (n≈372) are used for an HLA reference panel. Therefore, each of the 10 subgroups used exactly once as the test data in the cross-validation process. The classical HLA alleles in a test group were then imputed from MHC SNPs in the test group using SNP2HLA and the reference panel that was constructed from the matched reference group. Average concordance rates were calculated from concordance rates between imputed and actual 2-digit and 4-digit classical alleles (including deleted alleles in *HLA-DRB3*, *HLA-DRB4*, and *HLA-DRB5*) of each HLA gene. The average concordance rates between the best-guess imputed and actual 4-digit alleles of *HLA-DRB3*, *HLA-DRB4*, and *HLA-DRB5* were 91.7 to 98.4% (Table 1). The imputation accuracy for HLA-DRB1 was 89.6% at 2-digit resolution and 81.6% at 4-digit resolution, which was similar to that of previous Asian panels [3,5]. In addition, we checked the correlation of imputed dosage (0 to 2) with actual dosage (0, 1, or 2) for each HLA allele. For the pre-existing HLA variants in the original reference panel, a high correlation between imputed and actual dosage was observed (average Pearson’s correlation coefficient (*r*) = 0.909 for alleles with frequency ≥0.01), which was consistent with that of the original Korean HLA reference panel (*r* = 0.887 for alleles with frequency ≥0.01) [5]. Our new reference panel also showed good correlation coefficients for the alleles of *HLA-DRB3*, *HLA-DRB4*, and *HLA-DRB5* (*r* = 0.891 for alleles with frequency ≥0.01; S1 Fig).

**Table 1:** Concordance rate between imputed and actual allele of HLA genes^a^.

Allelic resolution	HLA genes									
	*A*	*B*	*C*	*DRB1*	*DPB1*	*DQB1*	*DRB3*	*DRB4*	*DRB5*	Total
2-digit	0.970	0.916	0.970	0.892	0.966	0.954	0.919	0.959	0.979	0.947
4-digit	0.913	0.867	0.930	0.815	0.949	0.908	0.918	0.958	0.979	0.915

^a^ Imputation accuracy was examined by cross-validation comparing the imputed and actual genotypes of classical alleles of *HLA*-*A*, *HLA*-*B*, *HLA*-*C*, *HLA*-*DRB1*, *HLA*-*DPB1*, *HLA*-*DQB1*, *HLA*-*DRB3*, *HLA*-*DRB4* and *HLA*-*DRB5*, as described following. The Korean HLA reference panel subjects (n = 413) were randomly divided into 10 almost equal sized subgroups (n≈41). Of the 10 subgroups, a single subgroup (n≈41) is retained as the test group to be imputed for HLA variants, and the remaining 9 subgroups (n≈372) are used for an HLA reference panel. The classical HLA alleles in a test group were then imputed from MHC SNPs in the test group using SNP2HLA and the reference panel that was constructed from the matched reference group. Average concordance rates were calculated from concordance rates between imputed and actual 2-digit and 4-digit classical alleles (including deleted alleles in *HLA-DRB3*, *HLA-DRB4*, and *HLA-DRB5*) of each HLA gene.

The HLA reference panel is publicly available at https://sites.google.com/site/scbaehanyang/hla_panel.

**Fig 1:**
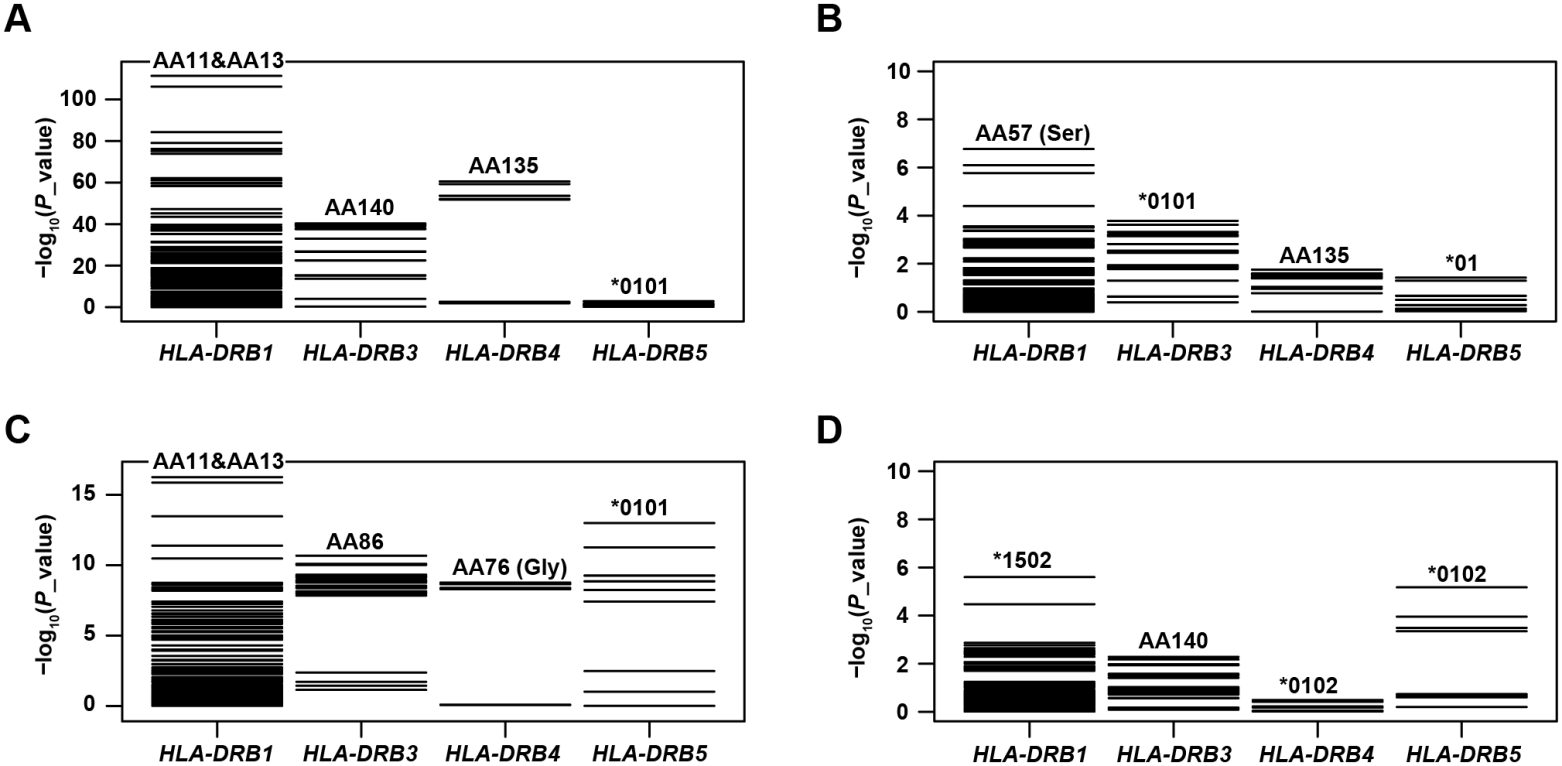
Distributions of p values for disease associations of HLA-DRB variants. P values for all variants of *HLA-DRB1*, *HLA*-*DRB3*, *HLA*-*DRB4* and *HLA*-*DRB5* were calculated by unconditional and conditional analyses testing associations with RA and SLE. The most significant association in unconditional analyses was identified at HLA-DRβ1 amino-acid position (AA) 11 and AA13 in both RA **(A)** and SLE **(C)**. After conditioning on all reported disease-associated amino-acid positions of HLA-DRβ1 (11, 13, 71, and 74 in RA and 11, 13, and 26 in SLE), no additional association with p < 5 × 10^−8^ was identified in RA **(B)** or SLE **(D)**. Variants with the lowest p value are shown for each HLA-DRB gene.

We then revisited our previous SNP datasets from Korean case-control cohorts for RA (9,271; 2,234 cases and 7,036 controls) [9] and SLE (5,342; 849 cases and 4,493 controls) [4] to examine the disease associations of all functional HLA-DRB genes. After imputation and association tests using logistic regression and log-likelihood ratio tests, we identified the primary disease association to be, among the HLA-DRB genes in the extended MHC region, at *HLA-DRB1*. The most significant association was identified at the two previously reported linked amino-acid positions, 11 and 13, of HLA-DRβ1 (at 11, p = 4.69 × 10^−112^ in RA and p = 5.64 × 10^−17^ in SLE) which accounted for disease risk better than any of the variants in the other HLA-DRB genes (p ≥ 3.62 × 10^−62^ in RA and p ≥ 1.03 × 10^−13^ in SLE) (Fig 1A and 1C). We note that the observed effects of the residues at these positions were consistent with previous reports [3,4].

To determine whether any secondary signals exist in the other HLA-DRB genes, we performed a conditional analysis controlling for *HLA-DRB1* association effects at the reported disease-associated amino-acid positions (positions 11, 13, 71, and 74 in RA; 11, 13, and 26 in SLE). There was no independent association in *HLA-DRB3*, *HLA-DRB4*, or *HLA-DRB5* passing the significance threshold p < 5 x 10^−8^ (Fig 1B and 1D).

In addition, we looked for haplotypic effects among *HLA-DRB* genes. It is well known that the presence of *HLA-DRB3*, *HLA-DRB4*, and *HLA-DRB5* perfectly correlates with two-digit alleles of *HLA-DRB1* [10–13]. For example, if the *HLA-DRB1* alleles *01, *08, or *10 are present, none of the other three HLA-DRB genes are found on the same chromosome phase. In contrast, *HLA-DRB3* is present whenever any of alleles *03, *11, *12, *13, or *14 of *HLA-DRB1* is. Similarly, *HLA-DRB4* is present whenever any of alleles *04, *07, or *09 of *HLA-DRB1* is, and *HLA-DRB5* is present whenever any of alleles *15 and *16 of *HLA-DRB1* is. When we obtained haplotypes of 4-digit classical alleles of the HLA-DRB genes, we were also able to observe known haplotype structures (*HLA-DRB1* alone, *HLA-DRB1*+*HLA-DRB3*, *HLA-DRB1*+*HLA-DRB4*, and *HLA-DRB1*+*HLA-DRB5*). The frequencies and disease association results of each haplotype in the RA and SLE case-control cohorts are shown in S1 and S2 Tables. However, we could not evaluate the modifying effects of *HLA-DRB3*, *HLA-DRB4*, or *HLA-DRB5* on the disease susceptibility effect of *HLA-DRB1*, because in most cases no classical allele of *HLA-DRB1* was tightly linked with more than two classical alleles of the other HLA-DRB genes (S1 and S2 Tables).

The exclusive association of *HLA-DRB1* among the HLA-DRB genes with RA and SLE may reflect an important role of structural variations in the HLA-DRβ1 epitope-binding site in the recognition of autoantigens in RA and SLE. Alternatively, pathogenic effects of *HLA-DRB3*, *HLA-DRB4*, or *HLA-DRB5* could be very small, perhaps due to their relatively low expression or weak function (for example, low affinity to HLA-DRα), which might result in poor representation of disease-risk alleles in patients with RA or SLE and thus low statistical power to detect disease association of such HLA-DRB genes. *HLA-DRB3* and *HLA-DRB4* are in fact expressed to a much lower degree than is *HLA-DRB1*, although *HLA-DRB5* is highly expressed [14–16].

## Conclusion

Previously, comprehensive research on all the functional HLA-DRB genes was lacking, or limited by the high cost of genotyping (resulting in small study sizes) [13,14] and the lack of imputation methods for *HLA-DRB3*, *HLA-DRB4*, and *HLA-DRB5* [2–4], despite interest in the association of HLA-DR with RA and SLE. In the present study, we constructed an HLA reference panel to impute all functional HLA-DRB genes as well as five other MHC class I and II HLA genes. By applying this panel to large case-control cohorts for RA and SLE, we revealed that the risk effects at *HLA-DRB3*, *HLA-DRB4*, and *HLA-DRB5* were neither superior to nor independent of the HLA-DRβ1 amino-acid model in RA and SLE.

In summary, our results support the association of *HLA-DRB1* with RA and SLE, and provide a more complete picture to better understand a source of disease association in the HLA-DR locus.

## Supporting Information

S1 Fig Dosage correlation between imputed and genotyped alleles. Imputed dosages (0 to 2) of 2-digit alleles (red), 4-digit alleles (green), and copy number of *HLA-DRB3*, *HLA-DRB4*, *HLA-DRB5* were compared with the actual dosage (0, 1 or 2). The correlation coefficient between the imputed and actual dosages of each allele with allele frequency ≥ 0.01 was plotted according to its allele frequency.
(TIF)

S1 Table Results for association of *HLA-DRB* haplotypes with RA susceptibility. (PDF)

S2 Table Results for association of *HLA-DRB* haplotypes with SLE susceptibility. (PDF)
