# Supplementary figures and images for: Imputing Variants in HLA-DR Beta Genes Reveals That HLA-DRB1 Is Solely Associated with Rheumatoid Arthritis and Systemic Lupus Erythematosus
Source: PLoS One. 2016 Feb 26;11(2):e0150283. doi: 10.1371/journal.pone.0150283 (PMC4769216; doi:10.1371/journal.pone.0150283)

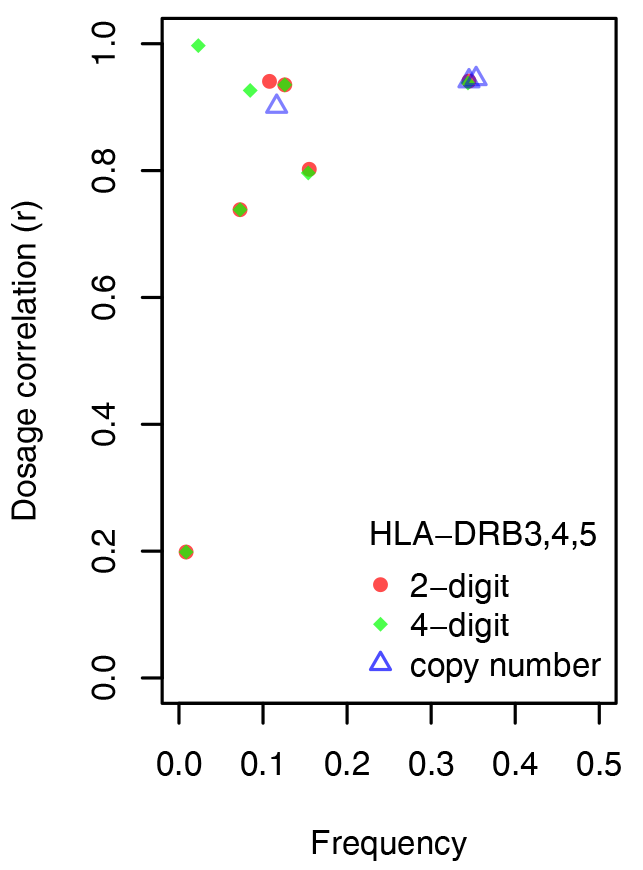

Supplement: S1 Fig — Imputed dosages (0 to 2) of 2-digit alleles (red), 4-digit alleles (green), and copy number of HLA-DRB3, HLA-DRB4, HLA-DRB5 were compared with the actual dosage (0, 1 or 2). The correlation coefficient between the imputed and actual dosages of each allele with allele frequency ≥ 0.01 was plotted according to its allele frequency. (TIF) [file pone.0150283.s001.tif]
