# Supplementary material for: Imputing Variants in HLA-DR Beta Genes Reveals That HLA-DRB1 Is Solely Associated with Rheumatoid Arthritis and Systemic Lupus Erythematosus
Source: PLoS One. 2016 Feb 26;11(2):e0150283. doi: 10.1371/journal.pone.0150283 (PMC4769216; doi:10.1371/journal.pone.0150283)
Supplement: S1 Table — (PDF) [file pone.0150283.s002.pdf]

S1 Table. Results for association of *HLA-DRB* haplotypes with RA susceptibility

DRB-allele haplotype*	RA; n (%)	Control; n (%)	OR (95% CI)	P
DRB1*0101	319 (7.14)	847 (6.02)	1.09 (0.89-1.34)	0.406
DRB1*0301 - DRB3*0202	48 (1.07)	276 (1.96)	0.73 (0.52-1.01)	0.060
DRB1*0403 - DRB4*0101	202 (4.52)	452 (3.21)	1.47 (1.22-1.77)	4.53E-05
DRB1*0405 - DRB4*0101	1069 (23.93)	1356 (9.63)	3.26 (2.87-3.70)	2.47E-73
DRB1*0406 - DRB4*0101	166 (3.72)	868 (6.16)	0.67 (0.53-0.84)	5.09E-04
DRB1*0410 - DRB4*0101	37 (0.83)	55 (0.39)	1.42 (0.92-2.20)	0.113
DRB1*0701 - DRB4*0101	185 (4.14)	929 (6.6)	0.38 (0.29-0.49)	6.76E-13
DRB1*0802	49 (1.1)	370 (2.63)	0.45 (0.33-0.61)	4.19E-07
DRB1*0803	253 (5.66)	948 (6.73)	0.67 (0.57-0.78)	5.90E-07
DRB1*0901 - DRB4*0101	587 (13.14)	1415 (10.05)	1.48 (1.31-1.66)	4.93E-11
DRB1*1001	171 (3.83)	217 (1.54)	2.69 (2.14-3.37)	2.07E-17
DRB1*1101 - DRB3*0202	197 (4.41)	839 (5.96)	0.72 (0.61-0.85)	1.19E-04
DRB1*1201 - DRB3*0101	116 (2.6)	586 (4.16)	0.74 (0.60-0.92)	5.74E-03
DRB1*1202 - DRB3*0301	137 (3.07)	427 (3.03)	0.98 (0.80-1.20)	0.826
DRB1*1301 - DRB3*0101	16 (0.36)	204 (1.45)	0.28 (0.17-0.47)	1.78E-06
DRB1*1302 - DRB3*0301	221 (4.95)	1343 (9.54)	0.59 (0.47-0.74)	2.81E-06
DRB1*1401 - DRB3*0202	90 (2.01)	498 (3.54)	0.53 (0.42-0.67)	1.32E-07
DRB1*1403 - DRB3*0101	47 (1.05)	178 (1.26)	0.56 (0.40-0.78)	6.30E04
DRB1*1405 - DRB3*0202	42 (0.94)	350 (2.49)	0.41 (0.29-0.57)	1.16E-07
DRB1*1501 - DRB5*0101	238 (5.33)	952 (6.76)	0.77 (0.66-0.90)	9.86E-04
DRB1*1502 - DRB5*0101	10 (0.22)	33 (0.23)	0.87 (0.42-1.79)	0.698
DRB1*1502 - DRB5*0102	126 (2.82)	446 (3.17)	1.35 (1.02-1.79)	0.036
DRB1*1602 - DRB5*0202	22 (0.49)	49 (0.35)	0.97 (0.58-1.63)	0.904

* Haplotypes with frequency > 0.1% in control groups are shown. Note: if the *HLA-DRB1* alleles *01, *08, or *10 were present, none of the other three DRB genes were present on the same chromosome phase. In contrast, *HLA-DRB3* was present whenever alleles *03, *11, *12, *13, or *14 of *HLA-DRB1* were present; *HLA-DRB4* was present whenever alleles *04, *07, or *09 of *HLA-DRB1* were present; and *HLA-DRB5* was present whenever alleles *15 or *16 of *HLA-DRB1* were present. Please note that association results for low-frequency haplotypes (<1%) could be unreliable.

RA: rheumatoid arthritis; OR: odds ratio; CI: confidence interval.
