# Supplementary material for: Imputing Variants in HLA-DR Beta Genes Reveals That HLA-DRB1 Is Solely Associated with Rheumatoid Arthritis and Systemic Lupus Erythematosus
Source: PLoS One. 2016 Feb 26;11(2):e0150283. doi: 10.1371/journal.pone.0150283 (PMC4769216; doi:10.1371/journal.pone.0150283)
Supplement: S2 Table — (PDF) [file pone.0150283.s003.pdf]

S2 Table. Results for association of *HLA-DRB* haplotypes with SLE susceptibility

DRB-allele haplotype*	SLE; n (%)	Control; n (%)	OR (95% CI)	P
DRB1*0101	81 (4.77)	576 (6.41)	0.73 (0.58-0.93)	9.98E-03
DRB1*0301 - DRB3*0202	45 (2.65)	168 (1.87)	1.43 (1.02-2.00)	0.039
DRB1*0403 - DRB4*0101	39 (2.3)	302 (3.36)	0.68 (0.49-0.96)	0.027
DRB1*0405 - DRB4*0101	127 (7.48)	802 (8.92)	0.82 (0.67-1.00)	0.046
DRB1*0406 - DRB4*0101	45 (2.65)	490 (5.45)	0.47 (0.35-0.65)	2.76E-06
DRB1*0410 - DRB4*0101	32 (1.88)	136 (1.51)	1.30 (0.88-1.92)	0.194
DRB1*0701 - DRB4*0101	165 (9.72)	596 (6.63)	1.53 (1.27-1.83)	6.17E-06
DRB1*0802	48 (2.83)	212 (2.36)	1.21 (0.88-1.67)	0.237
DRB1*0803	176 (10.37)	636 (7.08)	1.50 (1.26-1.79)	5.30E-06
DRB1*0901 - DRB4*0101	205 (12.07)	918 (10.22)	1.21 (1.04-1.43)	0.017
DRB1*1001	25 (1.47)	154 (1.71)	0.85 (0.55-1.30)	0.443
DRB1*1101 - DRB3*0202	65 (3.83)	513 (5.71)	0.66 (0.51-0.86)	1.89E-03
DRB1*1201 - DRB3*0101	53 (3.12)	295 (3.28)	0.94 (0.70-1.27)	0.692
DRB1*1202 - DRB3*0301	22 (1.3)	279 (3.1)	0.40 (0.26-0.63)	4.98E-05
DRB1*1301 - DRB3*0101	30 (1.77)	148 (1.65)	1.07 (0.72-1.60)	0.725
DRB1*1302 - DRB3*0301	105 (6.18)	826 (9.19)	0.66 (0.53-0.81)	1.07E-04
DRB1*1401 - DRB3*0202	55 (3.24)	340 (3.78)	0.85 (0.63-1.13)	0.258
DRB1*1403 - DRB3*0101	27 (1.59)	235 (2.62)	0.59 (0.40-0.89)	0.011
DRB1*1405 - DRB3*0202	40 (2.36)	217 (2.41)	0.98 (0.70-1.37)	0.916
DRB1*1501 - DRB5*0101	218 (12.84)	680 (7.57)	1.82 (1.54-2.14)	1.10E-12
DRB1*1502 - DRB5*0101	10 (0.59)	20 (0.22)	2.75 (1.28-5.92)	9.66E-03
DRB1*1502 - DRB5*0102	42 (2.47)	321 (3.57)	0.67 (0.49-0.93)	0.016
DRB1*1602 - DRB5*0202	27 (1.59)	80 (0.89)	1.75 (1.13-2.72)	0.013

* Haplotypes with frequency > 0.1% in control groups are shown. Note: if the *HLA-DRB1* alleles *01, *08, or *10 were present, none of the other three DRB genes were present on the same chromosome phase. In contrast, *HLA-DRB3* was present whenever alleles *03, *11, *12, *13, or *14 of *HLA-DRB1* were present; *HLA-DRB4* was present whenever alleles *04, *07, or *09 of *HLA-DRB1* were present; and *HLA-DRB5* was present whenever alleles *15 or *16 of *HLA-DRB1* were present. Please note that association results for low-frequency haplotypes (<1%) could be unreliable.

SLE: systemic lupus erythematosus; OR: odds ratio; CI: confidence interval.
